# A pathway for multivariate analysis of ecological communities using copulas

**DOI:** 10.1002/ece3.4948

**Published:** 2019-03-05

**Authors:** Marti J. Anderson, Perry de Valpine, Andrew Punnett, Arden E. Miller

**Affiliations:** ^1^ New Zealand Institute for Advanced Study (NZIAS) Massey University Auckland New Zealand; ^2^ PRIMER‐e (Quest Research Limited) Auckland New Zealand; ^3^ Department of Environmental Science, Policy and Management University of California Berkeley California; ^4^ Department of Statistics University of Auckland Auckland New Zealand

**Keywords:** abundance data, discrete counts, over‐dispersion, species’ associations, statistical model, zero‐inflation

## Abstract

We describe a new pathway for multivariate analysis of data consisting of counts of species abundances that includes two key components: copulas, to provide a flexible joint model of individual species, and dissimilarity‐based methods, to integrate information across species and provide a holistic view of the community. Individual species are characterized using suitable (marginal) statistical distributions, with the mean, the degree of over‐dispersion, and/or zero‐inflation being allowed to vary among a priori groups of sampling units. Associations among species are then modeled using copulas, which allow any pair of disparate types of variables to be coupled through their cumulative distribution function, while maintaining entirely the separate individual marginal distributions appropriate for each species. A Gaussian copula smoothly captures changes in an index of association that excludes joint absences in the space of the original species variables. A permutation‐based filter with exact family‐wise error can optionally be used a priori to reduce the dimensionality of the copula estimation problem. We describe in detail a Monte Carlo expectation maximization algorithm for efficient estimation of the copula correlation matrix with discrete marginal distributions (counts). The resulting fully parameterized copula models can be used to simulate realistic ecological community data under fully specified null or alternative hypotheses. Distributions of community centroids derived from simulated data can then be visualized in ordinations of ecologically meaningful dissimilarity spaces. Multinomial mixtures of data drawn from copula models also yield smooth power curves in dissimilarity‐based settings. Our proposed analysis pathway provides new opportunities to combine model‐based approaches with dissimilarity‐based methods to enhance understanding of ecological systems. We demonstrate implementation of the pathway through an ecological example, where associations among fish species were found to increase after the establishment of a marine reserve.

## 1 INTRODUCTION

Multivariate ecological community data, consisting of counts of species’ abundances, have a number of salient statistical properties that have been studied over many decades (Bliss & Fisher, 1953; ter Braak, 1996; Clarke, Chapman, Somerfield, & Needham, 2006; Martin et al., 2005; McArdle, Gaston, & Lawton, 1990; Taylor, Woiwood, & Perry, 1979; Whittaker, 1952). They are typically high dimensional (Dunstan, Foster, Hui, & Warton, 2013)—the number of species (*p*) often exceeds the number of sampling units (*N*), and most species are rare (McGill et al., 2007), contributing many zeros to an (*N* × *p*) matrix of count data (**Y**). In addition, individual variables generally show over‐dispersion (McArdle et al., 1990; White & Bennetts, 1996) and often zero‐inflation (Martin et al., 2005; Welsh, Cunningham, Donnelly, & Lindenmayer, 1996; Wenger & Freeman, 2008). The degree of over‐dispersion and zero‐inflation varies, not only among species (Clarke, Chapman et al., 2006; Taylor, 1961), but also within a species across different environmental conditions, temporally or spatially (McArdle & Anderson, 2004; Smith, Anderson, & Millar, 2012; Taylor et al., 1979). Thus, multi‐species datasets consist of mixed variable types; counts of different species are generally incommensurable, due to differences in the sizes, morphologies, life‐history strategies, detectabilities, and behaviors of different species.

Species also display associations with one another (Somerfield & Clarke, 2013). Statistical non‐independence may reflect phylogenetic or functional inter‐relationships (Paradis & Claude, 2002), synchronous (or asynchronous) behavior or dispersal mechanisms (Kendall, Bjørnstad, Bascompte, Keitt, & Fagan, 2000), or inter‐specific interactions, such as competition (Goldberg & Landa, 1991), commensalism, or trophic relationships (Zurell, Pollock, & Thuiller, 2018). Associations can also be generated indirectly through species responding in similar (or opposing) ways to environmental gradients or habitats (Dunstan, Foster, & Darnell, 2011; Warton et al., 2015). Furthermore, relationships among species vary in time and space under changing biotic or abiotic conditions (Clark, Wells, & Lindberg, 2018); for example, species may only compete when resources are limiting (Perry, Mitchell, Zutter, Glover, & Gjerstad, 1994), in certain parts of their range (Pacala & Roughgarden, 1985), or in the absence of predation (Chase et al., 2002).

To analyze multi‐species count data, many researchers have used methods based on an *N* × *N* matrix of dissimilarities, **D**, among sampling units (Anderson, 2001; Clarke, 1993). These approaches handle high‐dimensional count data and reliably detect important changes in the structure of ecological communities (Anderson, 2001; Clarke, 1993; Clarke, Somerfield, & Chapman, 2006; Legendre & De Cáceres, 2013). The focus here is to measure holistic changes in the identities of species and potentially also changes in species’ relative or proportional abundances. Dissimilarities, often calculated using measures that exclude joint absences, such as Bray–Curtis, Hellinger, or Jaccard (Legendre & Legendre, 2012), integrate information across all species to define ecological relationships among sampling units. Some measures (such as Gower's measure, 1971) also accommodate data having different types of variables (see Legendre & Legendre, 2012). Most dissimilarity measures of interest to ecologists emphasize the extent to which two sampling units either share, or do not share, any species in common. Thus, important community‐level concepts such as beta diversity (Anderson et al., 2011; Vellend, 2001) and turnover (Baselga, 2010), for example, are measured using dissimilarities. However, dissimilarity‐based methods create no formal model of the original variables. Roles of individual species and relationships among them are not directly identifiable, as no species‐specific parameters are estimated. Hence, one cannot predict the makeup of communities under defined scenarios, nor readily calculate power.

To characterize ecological communities and make species‐level predictions under specified hypotheses, formal joint statistical models of the species variables are required. The multi‐faceted challenge for developing such models is to deal simultaneously with (generally) over‐dispersed, zero‐inflated, high‐dimensional, inter‐related, mixed sets of (usually discrete) variables, including a host of rare species, for which no single multivariate statistical distribution can be readily articulated. A successful model of community data should allow a wide variety of species‐specific marginal distributions that can flexibly change in time and space, while accounting for meaningful inter‐specific associations.

There has been rapid recent development of new statistical models for multivariate species data that also incorporate inter‐specific associations (Clark, Nemergut, Seyednasrollah, Turner, & Zhang, 2017; Clark et al., 2018; Golding & Purse, 2016; Harris, 2015, 2016; Hui, 2016; Hui, Taskinen, Pledger, Foster, & Warton, 2015; Nieto‐Lugilde, Maguire, Blois, Williams, & Fitzpatrick, 2017; Niku, Warton, Hui, & Taskinen, 2017; Pollock et al., 2014; Popovic, Hui, & Warton, 2018; Thorson et al., 2016; Warton et al., 2015). Several of these, such as stochastic feed‐forward neural networks (“*mistnet*”; Harris, 2015), Bayesian Gaussian process models (GP SDMs; Golding & Purse, 2016), or Markov random fields (MRF; Clark et al., 2018), have so far been used only on presence–absence data to enhance species distribution models (SDMs, Elith & Leathwick, 2009), although extensions to abundance data may well be feasible.

Models of multivariate abundance data include generalized linear models (GLMs) with (typically, for counts) a log link and either a Poisson or negative binomial (NB) error, and with correlations between species modeled using generalized estimating equations (GEEs, Wang, Naumann, Wright, & Warton, 2012; Warton, 2011; Warton & Guttorp, 2011). Alternatively, relationships among species can be modeled parsimoniously by including latent random variables as linear predictors in the GLM—called generalized linear latent variable models (GLLVMs, Hui et al., 2015; Niku et al., 2017; Warton et al., 2015). The latent variables are intended to capture correlations due to unmeasured environmental drivers or biotic interactions, and one can use model selection to identify an appropriate number of latent variables to include in the model (Hui et al., 2015). More sophisticated GLLVMs can also accommodate spatial/temporal autocorrelation (Ovaskainen, Roy, Fox, & Anderson, 2016; Thorson et al., 2016, 2015), the inclusion of species’ traits/phylogenies (Ovaskainen et al., 2017), or variation in correlations at different hierarchical spatial scales (Ovaskainen, Abrego, Halme, & Dunson, 2016).

Generalized joint attribute models (GJAMs) have also been proposed for modeling multivariate ecological data (Clark et al., 2017). GJAMs model covariances between mixed variable types (presence–absence, ordinal, discrete, or continuous) on their original scales. Discrete data (such as counts) are modeled via censoring, with partitions and weights chosen to allow linkages between different variable types (Clark et al., 2017). Partition widths and associated effort in interval censoring also can be chosen arbitrarily to accommodate mean–variance relationships. Imputation across censored intervals maps discrete variables into a multivariate normal (MVN) space to estimate covariances, with Bayesian analysis being used to estimate the latent (imputed) states.

We consider that copulas (Mai & Scherer, 2017) also hold great promise for flexible joint modeling of multivariate ecological count data (Popovic et al., 2018). A copula is a function representing a joint distribution as a mapping from the cumulative distribution functions (cdf) of its marginals, hence can be used to couple virtually any pair of variables (Mai & Scherer, 2017; Sklar, 1959). Copulas allow a tailored multivariate distribution to be constructed from two separate parts: (a) the univariate marginal distributions for each variable; and (b) the joint distributions of the variables in a multivariate copula space. Although implemented fairly widely in other fields (Nikoloulopoulos & Karlis, 2009; Shi & Valdez, 2014), copulas have not yet been widely used in ecology (but see de Valpine, Scranton, Knape, Ram, & Mills, 2014; Popovic et al., 2018).

Our primary motivation for using copulas for ecological count data is that they allow any marginal distribution to be used for any variable. One does not need to forego the utility of the wide potential array of existing univariate statistical distributions, each with its own interpretable parameters, to build a joint model. Also, in copula models, associations among variables are modeled separately from their marginal distributions, making them easy to interpret, and the particular copula distribution used to model associations can also be flexibly chosen to fit a specific context. In contrast, latent variable models typically confound correlation structures with marginal distributions, as correlations among species are induced *via* latent variables that in turn alter the marginal distributions.

Here, we shall restrict our attention to Gaussian (MVN) copula distributions, and also to counts of species (abundance data) arising from one‐way ANOVA‐type designs, but the core ideas are readily extended to other types of mixed datasets, other copula distributions, and/or more complex sampling/experimental designs. Gaussian copulas can draw on the rich statistical literature surrounding MVN distributions, while tailoring marginal distributions to non‐normal ecological variables. Fortunately, difficulties in estimating parameters for Gaussian copulas with discrete marginals (Faugeras, 2017; Genest & Nešlehová, 2007) have recently been surmounted (see Appendix 1).

The aim of this work is twofold. First, we provide an accessible description of copulas and show how they can work for ecological count data through a simple bivariate example. Second, we outline a pathway for the analysis of multivariate ecological count data that combines the use of copulas with dissimilarity‐based methods. More specifically, copulas are first used to characterize the properties of individual variables and their associations in a formal parametric statistical model. Dissimilarity‐based methods are then used to examine community‐level patterns for whole assemblages of species that have been simulated from these copula models under defined scenarios.

Our analysis pathway (a) characterizes each individual species via estimation of marginal distributions and their associated parameters; (b) addresses high dimensionality (optionally) by screening data to identify significant pair‐wise associations, using an index that excludes joint absences; (c) characterizes associations among species via estimation of a copula model and its associated parameters; and (d) proposes simulation from copula models to generate realistic ecological data under specified null or alternative hypotheses for model‐based inference, ordination, and power analysis in dissimilarity‐based settings. In the proposed pathway, we allowed both the marginal parameters and the copula parameters to vary across a priori groups, to maximize flexibility.

**Figure 1:**
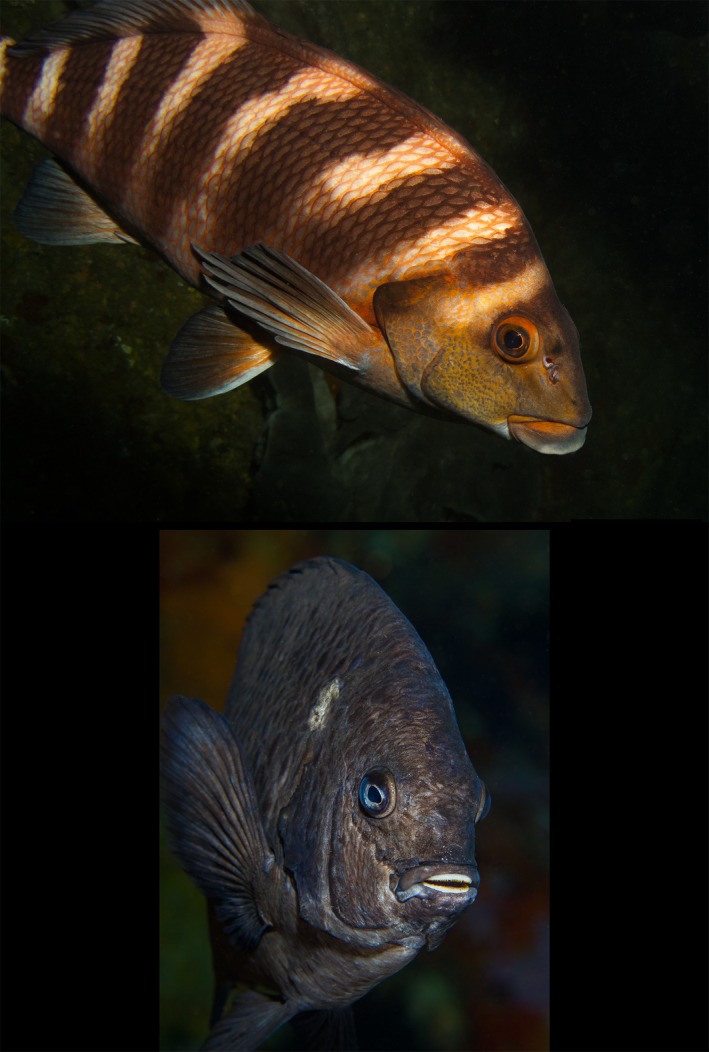
Two fish species found at the Poor Knights Islands, New Zealand: *Chirodactylus spectabilis* (top) and *Parma alboscapularis* (bottom). Photographs by Paul Caiger

We demonstrate the analysis pathway with an example dataset: counts of fishes (*p* = 47 species) from the Poor Knights Islands, New Zealand (two of the 47 species are shown in Figure 1). Sampling occurred at three different times (September 1998: *n*_1_ = 15, March 1999: *n*_2_ = 21, and September 1999: *n*_3_ = 20), spanning the establishment of a no‐take marine reserve in October 1998 (Willis & Denny, 2000; data are provided in Supporting Information Table S1 and are also available from the Dryad Digital Repository: https://doi.org/10.5061/dryad.3s6rm0f).

## 2 A GAUSSIAN COPULA FOR DISCRETE NON‐NORMAL DATA

A copula is defined by Sklar's seminal theorem (1959). Let *F* be a *p*‐dimensional distribution function with margins *F*_1_, *F*_2_, …, *F*_p_. There exists a *p*‐dimensional copula *C* such that for all (*y*_1_, *y*_2_, …, *y*_p_) ∈ ℝ _p_ the following holds:

(1) $$F\left(y_{1},\;y_{2},\ldots ,\;y_{\text{p}}\right)=C\left(F_{1}\left(y_{1}\right),\;F_{2}\left(y_{2}\right),\ldots ,\;F_{\text{p}}\left(y_{\text{p}}\right)\right)$$

*C* is unique if *F*_1_, *F*_2_, …, *F*_p_are continuous. Conversely, if *C*is a *p*‐dimensional copula and* F*_1_, *F*_2_, …, *F*_p_are univariate distribution functions, then the function *F*(*y*_1_, *y*_2_, …, *y*_p_) is a *p*‐dimensional distribution function (Mai & Scherer, 2017).

**Figure 2:**
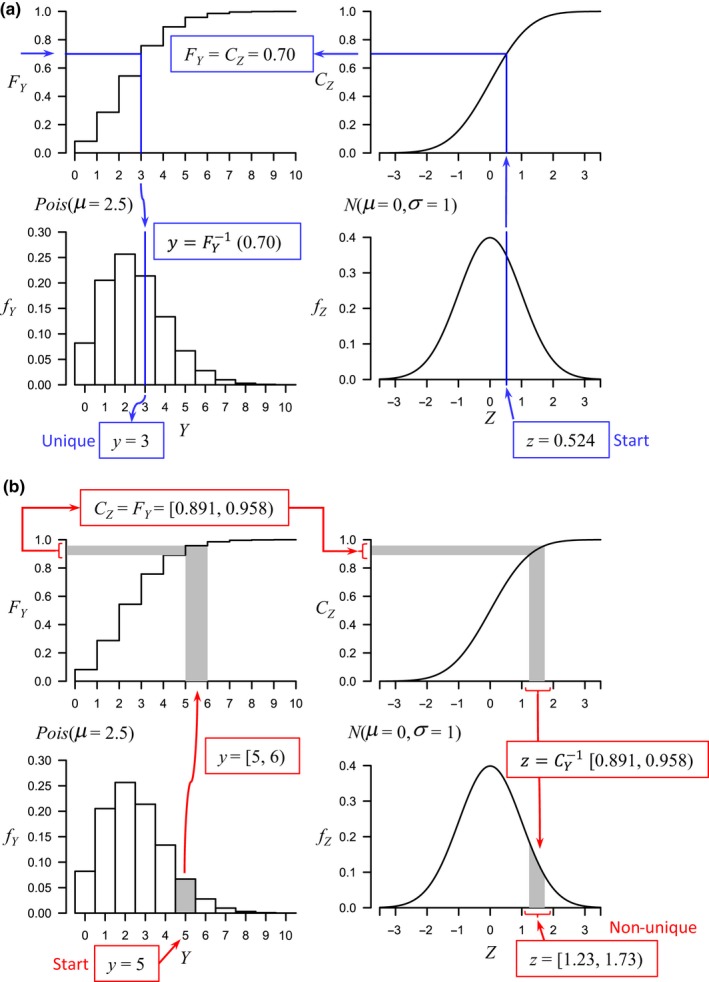
Schematic diagram showing the mapping between the probability density function of a continuous variable and the probability mass function of a discrete variable via their respective cumulative distribution functions (cdfs); (a) the mapping from the continuous to the discrete yields a unique value; (b) the mapping from the discrete to the continuous is non‐unique, but produces a range of values in the space of the continuous variable

To understand how copulas work, consider that the position of an individual value *y* of a random variable *Y* having probability density function (pdf) *f*_Y_(*y*) is able to be expressed as a value along its cdf, denoted *F*_Y_(*y*), on the interval [0,1]. This provides a direct mapping of values from one pdf to another via their cdfs. This approach can also be used to map a value drawn from the pdf of a continuous random variable to the probability mass function (pmf) of a discrete random variable (Figure 2a). For example, consider a random variable *Y* ~ Poisson (*μ* = 2.5) and a standard normal variable *Z* ~ *N*(*μ* = 0, *σ* ^2^ = 1), whose cdf we will denote by *C*_Z_(*z*). A random value *z* drawn from *Z* can be mapped on to *Y* uniquely by taking the inverse function: $y=F_{\text{Y}}^{-1}\left(C_{\text{Z}}\left(z\right)\right)$. Thus, suppose we draw *z* = 0.524, then *C*_Z_ (0.524) = 0.70; that is, we have drawn the 70th percentile of *f*_Z_(*z*). Mapping *F*_Y_(*y*) one‐to‐one on *C*_Z_(*z*), the 0.70 quantile of *f*_Y_(*y*) is then given by $y=F_{\text{Y}}^{-1}\left(0.70\right)=3$ (Figure 2a, blue arrows).

**Figure 3:**
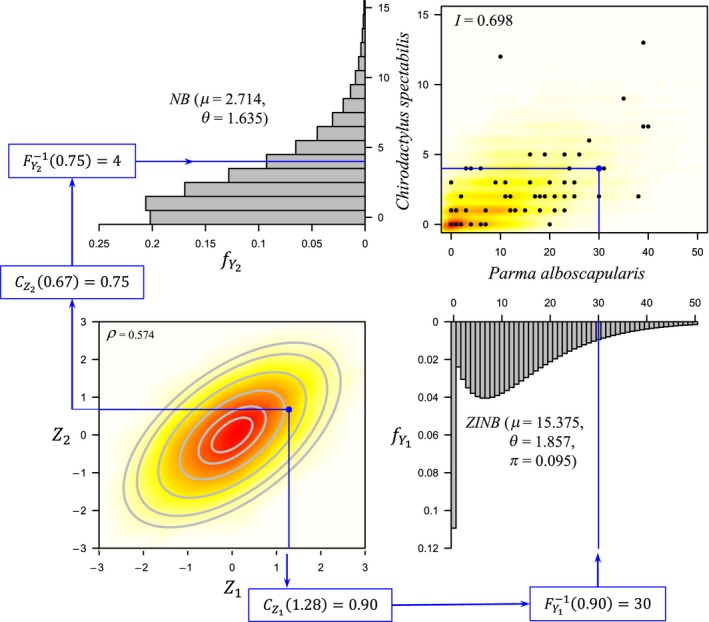
Standard bivariate normal (Gaussian) copula model with discrete marginal distributions for two fish species from the Poor Knights Islands. Points drawn from the copula space with correlation parameter *ρ* = 0.574 generate, through the specified negative binomial (NB) and zero‐inflated NB marginal distributions, points in the discrete bivariate data space of counts that correspond to an expected inter‐specific index of association of *I* = 0.698

This simple mapping idea is exploited for modeling the joint distribution (association) between any pair of disparate types of variables in a copula. Here, we shall use a Gaussian copula to handle multiple associated variables simultaneously (but see Mai and Scherer (2017); Shi and Valdez (2014) for a broader variety of copula distributions). Consider a bivariate ecological example, where *Y*_1_ are counts of individual fish of the species *Chirodactylus spectabilis* (red moki), and *Y*_2_ are counts of *Parma alboscapularis* (black angelfish) from the Poor Knights Islands (Figure 1). Model selection (using AICc) performed separately on each variable for the full set of data (*N* = 56) suggested the NB (*μ* = 2.714, *θ* = 1.635) and the zero‐inflated NB (ZINB; *μ* = 15.375, *θ* = 1.857, *π* = 0.095) are suitable marginal distributions to model *Y*_1_ and *Y*_2_, respectively (Figure 3). (Note: we have simply posited here that the parameters for each of these marginal distributions are equivalent to their maximum likelihood estimates.)

A measure of association between two species that excludes joint absences is the index of association (Somerfield & Clarke, 2013; Whittaker, 1952):

$$I_{k\ell }=1-\frac{1}{2}\sum_{i=1}^{N}\left|\left(\frac{y_{ik}}{\sum_{j=1}^{N}y_{jk}}\right)-\left(\frac{y_{i\ell }}{\sum_{j=1}^{N}y_{j\ell }}\right)\right|$$

where $y_{ik}$ denotes the count for species k in sampling unit *i* and *I* $I_{k\ell }$ denotes the association between species k and species ℓ. For red moki and black angelfish, there is a statistically significant positive association of *I* = 0.698 (*p* = 0.0001, 10,000 permutations).

**Figure 4:**
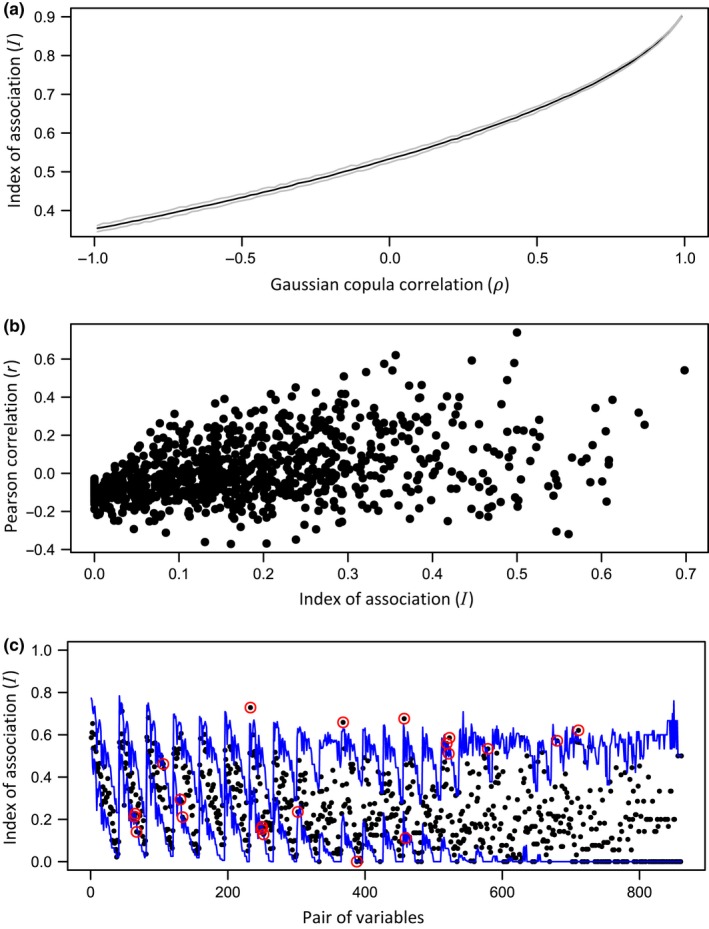
(a) Relationship between the index of association (*I*) in the discrete bivariate data space, with marginal distributions of negative binomial (NB) (*μ* = 2.714, *θ* = 1.635) and zero‐inflated NB (*μ* = 15.375, *θ* = 1.857, *π* = 0.095; see Figure 3), as a smooth function of the correlation parameter (*ρ*) in the standard bivariate normal (Gaussian) copula space. The black line follows the mean and the gray lines follow the upper 0.975 and lower 0.025 quantiles of the distribution of 100 values of *I* that were each calculated using a sample of 5,000 simulated datasets from the multivariate normal copula distribution at each value of *ρ* (taken at 0.02‐unit intervals between −1.0 and 1.0). (b) Relationship between the Pearson correlation (*r*) and the index of association (*I*) for all pairs of 47 variables (counts of fish species) from the Poor Knights Islands. (c) Index of association (*I*) between every pair of variables (black dots) for the fish data from the Poor Knights Islands for Time 2 only (*p* = 42, as five species did not occur at Time 2). Pairs are shown along the *x*‐axis in decreasing order of species’ importance, defined as frequency of occurrence. Blue lines show the upper and lower bounds from the permutation distribution of *I*, specific to each pair, for a two‐tailed per‐comparison empirical error rate of 0.01, obtained using 99,999 permutations. Red circles identify statistically significant associations

To model this association, we can use a standard bivariate normal distribution for the copula function (with variables *Z*_1_ and *Z*_2_) having correlation parameter *ρ* = 0.574 (lower left panel, Figure 3). A random sample of **z** = (1.28, 0.67) in the copula space (Figure 3, lower left) can be mapped into the bivariate space of species count data (Figure 3, upper right) by taking the inverse of the corresponding cdf values (the 90th and the 75th percentile, respectively) on each marginal distribution, that is, $y=\left(F_{Y_{1}}^{-1}\left(0.90\right),F_{Y_{2}}^{-1}\left(0.75\right)\right)$, hence ***y*** = (30, 4). A large number of such random draws from the copula model will generate count data in the species space that preserves their association as well as their individual (and disparate) marginal distributions (Figure 3). For a given pair of variables, there is a smooth monotonic relationship between rho (*ρ*) in the copula space and the index of association (*I*) in the space of the original variables (Figure 4a), highlighting the utility of Gaussian copulas in ecological research. In contrast, Pearson correlations (*r*) calculated among the original variables do not show a strong relationship with the index of association (Figure 4b), as the former do not omit joint‐absence information (Somerfield & Clarke, 2013).

One important complication, however, is the fact that a single point in the discrete data space corresponds to an entire region (a hyper‐rectangle) in the copula space (Figure 2b). Thus, estimation of the copula parameter(s) from discrete datasets is problematic, as the mapping of discrete values into the copula space is non‐unique. We can address this by performing Monte Carlo integration over each discrete interval (Shi & Valdez, 2014). Computational efficiency is achieved through a Monte Carlo expectation maximization (MCEM) algorithm (Wei & Tanner, 1990). Estimation of the parameters of the correlation matrix for a Gaussian copula with discrete marginal distributions is described in greater detail in Appendix 1.

## 3 A COPULA MODEL FOR ECOLOGICAL COUNT DATA

**Figure 5:**
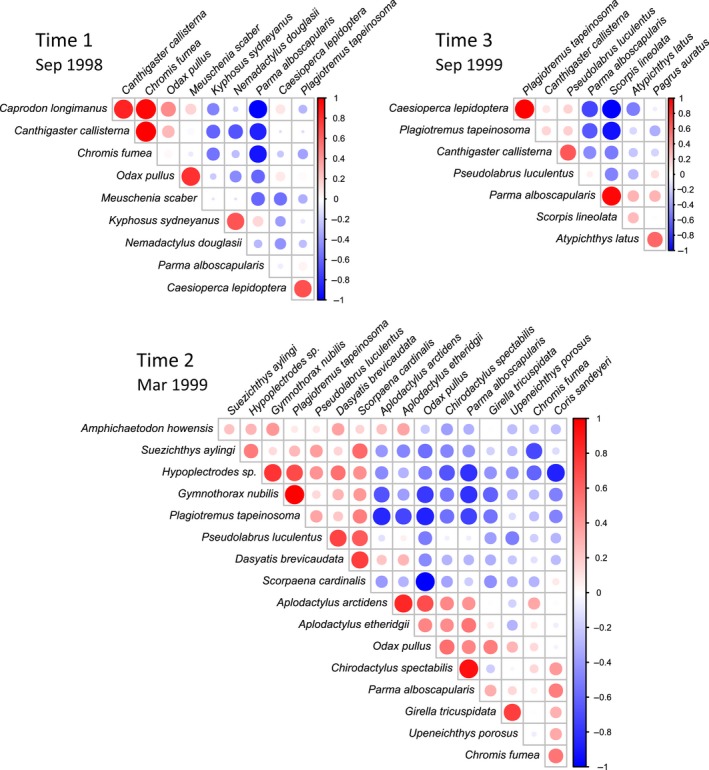
Heat maps of estimated copula correlation parameters for samples obtained at each of three different times (September 1998, March 1999, and September 1999) among fish species from the Poor Knights Islands that showed at least one statistically significant index of association (using a per‐comparison error rate of 0.01 as a filter, e.g., see Figure 4c)

We propose the following steps to develop a full copula‐based model for high‐dimensional ecological count data:

- *Identify appropriate marginal distributions*. For each species, an information criterion (such as AIC or AICc) may be used to choose among potential marginal distributions. Here, we restricted our attention to the following count distributions: Poisson, ZIP, NB, or ZINB (see Supporting Information Data S1 for R code). This step may include, for efficiency, identification of rare species that do not contain enough information to allow estimation of associations. Species may be flagged as “rare” if they occur as singletons or in only a small percentage of sampling units (e.g., <5%). We used AICc to identify marginal distributions for each of the *p* = 47 fish species in the Poor Knights dataset (Supporting Information Table S1), allowing both the statistical distribution and estimated parameters to vary across the three groups.
- *Identify significant associations among species to model*. An index of association is calculated between every pair of species, the null hypothesis of no association is tested for each pair using *P*‐values obtained by permutations (Somerfield & Clarke, 2013), and the significance level for the tests is suitably adjusted for multiple tests (see Supporting Information Data S2 for R code). For efficiency, species flagged as “rare” may (optionally) be omitted. For the adjustment, one may use, for example, an exact family‐wise error rate (FWER), empirically derived from the full set of permutation distributions (Wheldon, Anderson, & Johnson, 2007), or a more conservative per‐comparison error rate (PCER). We used a PCER of 0.01 for the fish data. This was done separately for each group. We identified five significant associations (involving 10 species) in September 1998, followed by a sharp increase to 20 associations (involving 17 species) in March 1999 (Figure 4c), and subsequent decrease to five associations (involving eight species) in September 1999. This step acts as a filter to reduce the size of the estimation problem for building a copula model and omits joint absences in the assessment of species’ associations. It is, however, optional; one could allow the copula model in step (iii) to include all inter‐specific associations.
- *Build a copula model and estimate its parameters*. We may reduce the problem to a subset of species, *m* ≤ *p*, showing a significant association with any other species. Given this subset of *m* species identified in step (ii), and each of their marginal distributions from step (i), estimate the parameters of the Gaussian copula's correlation matrix (see Appendix 1 for details; R code is provided in Supporting Information Data S3). We condition the estimation of copula correlation parameters on the fixed marginal distributions, which is both practical (ensuring marginal distributions fit each individual variable well) and efficient (Joe, 2005). Here, we estimated a separate copula correlation matrix for each group. Note that we may have chosen not to implement step (ii) above, or perhaps, even though step (ii) may reduce dimensionality dramatically (*m* ≪ *p*), we may still have *N* < *m*, or *m* may begin to approach *N* such that some form of regularization is still desirable. In such cases, as we are using Gaussian copulas, a variety of methods for regularizing the inverse covariance matrix may be considered (Friedman, Hastie, & Tibshirani, 2008; Schäfer & Strimmer, 2005; Ullah & Jones, 2015; Yuan & Lin, 2007); for simplicity, we shall not pursue the topic of regularization further here. Furthermore, we hasten to add that neither rare species nor unassociated species are omitted from the copula models that follow, but they are presumed to be independent of other species. For the fish dataset, species’ associations varied through time, and structured groups of associated species were easily seen in copula correlation matrices (Figure 5). There was an increase in the strength of associations after the establishment of the marine reserve (March 1999), which later subsided (September 1999). This is unlikely to have been a seasonal effect, as only three of the 10 species that showed significant associations in September 1998 did so in the following September 1999 (Figure 5).

## 4 SIMULATE DATA AND VISUALIZE RESULTS

**Figure 6:**
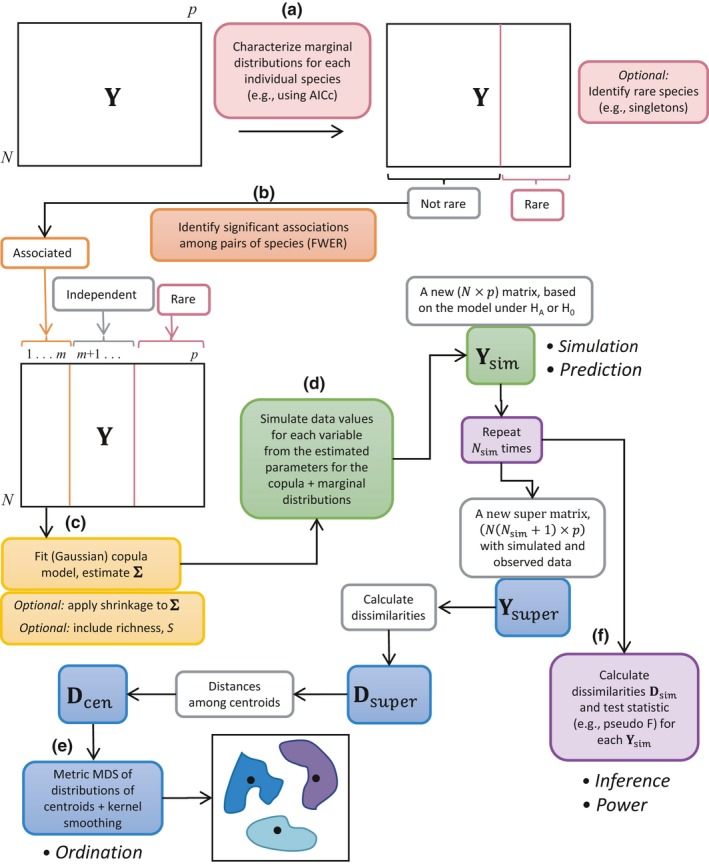
Schematic diagram of an overall pathway for analyzing ecological community data consisting of the following steps: (a) characterize marginal distributions for each species; (b) identify associations to model; (c) fit the copula; (d) simulate data under the full copula‐based model for predictions under null or alternative hypotheses; (e) visualize dissimilarities among sampling units or centroids under the model using robust ordination techniques; and (f) calculate power for test statistics of interest

An overall pathway to model, simulate, and visualize ecological community data using copulas is shown schematically in Figure 6. Once the parameters for all of the marginal distributions and the correlation matrix of the copula have been estimated for a given group (Figure 6a–c), then one can readily draw a random sample of a given modeled community, ***y***_sim_ (a vector of length *p*), for that group (Figure 6d) as follows:

1. Suppose there are *i* = 1, …, *g* groups. Let the subset number of species in group *i* showing a significant association with any other species in that group be denoted by *m_i_* and their estimated (*m_i_* × *m_i_*) correlation matrix in the Gaussian copula be denoted by ${\hat{\Sigma }}_{\left[m_{i}\right]}$. Expand this to obtain a (*p* × *p*) correlation matrix ${\hat{\Sigma }}_{i}$ for group *i* by placing zeros in the remaining off‐diagonal elements (corresponding to species that will be modeled as independent of one another), and 1s along the remaining diagonal elements.
2. Draw a random sample (a vector of length *p*) from the *p*‐dimensional Gaussian copula distribution whose correlation matrix is ${\hat{\Sigma }}_{i}$. Map the values obtained for each dimension in the copula space through the cdf of the individual marginal distribution for each species to obtain a simulated count value for each of the *p* species in the community.

Ordination plots of simulated data with original data can be used as a simple diagnostic to assess how sensible the model may be. What is usually of greater interest, however, is to consider changes in community structure among groups in the high‐dimensional space articulated by the model. For this, we desire an ordination of the group centroids, along with some measure of the expected variation in those centroids (based on the model). This can be examined on the basis of any dissimilarity measure of interest.

Suppose there are *n_i_* sampling units in group *i* and $N=\sum_{i=1}^{g}n_{i}$. One generates a new (*N* × *p*) matrix of simulated data **Y**_sim_ under the full copula model by drawing the *n_i_* sampling units, consisting of *p*‐length vectors ***y***_sim_, for each group in accordance with that group's estimated copula correlation matrix ${\hat{\Sigma }}_{i}$, marginal distributions, and associated parameters. This is then repeated *N*_sim_times (where *N*_sim_ is typically somewhat large, say *N*_sim_ = 100). One can then construct a super‐matrix **Y**_super_of dimension (*N*(*N*_sim_ + 1) × *p*) which (row‐wise) stacks the original matrix (**Y**) together with all of the **Y**_sim_ matrices obtained *via* simulation under the model. From this, a chosen dissimilarity measure is calculated to yield **D**_super_. We wish to map the *N*_sim_ × *g* centroids for all of the groups from every simulated dataset along with the *g* original centroids onto an ordination diagram. We can do this by calculating the distances among the (*N*_sim_ + 1) × *g* centroids from the **D**_super_ matrix directly (Anderson, 2017) to obtain **D**_cen_. Metric multi‐dimensional scaling (mMDS) can be used to visualize the distributions of centroids for each group under the model, along with the original group centroids. Kernel density contours (Duong, 2007) clarify the shapes of these distributions in the ordination space (Figure 6e).

**Figure 7:**
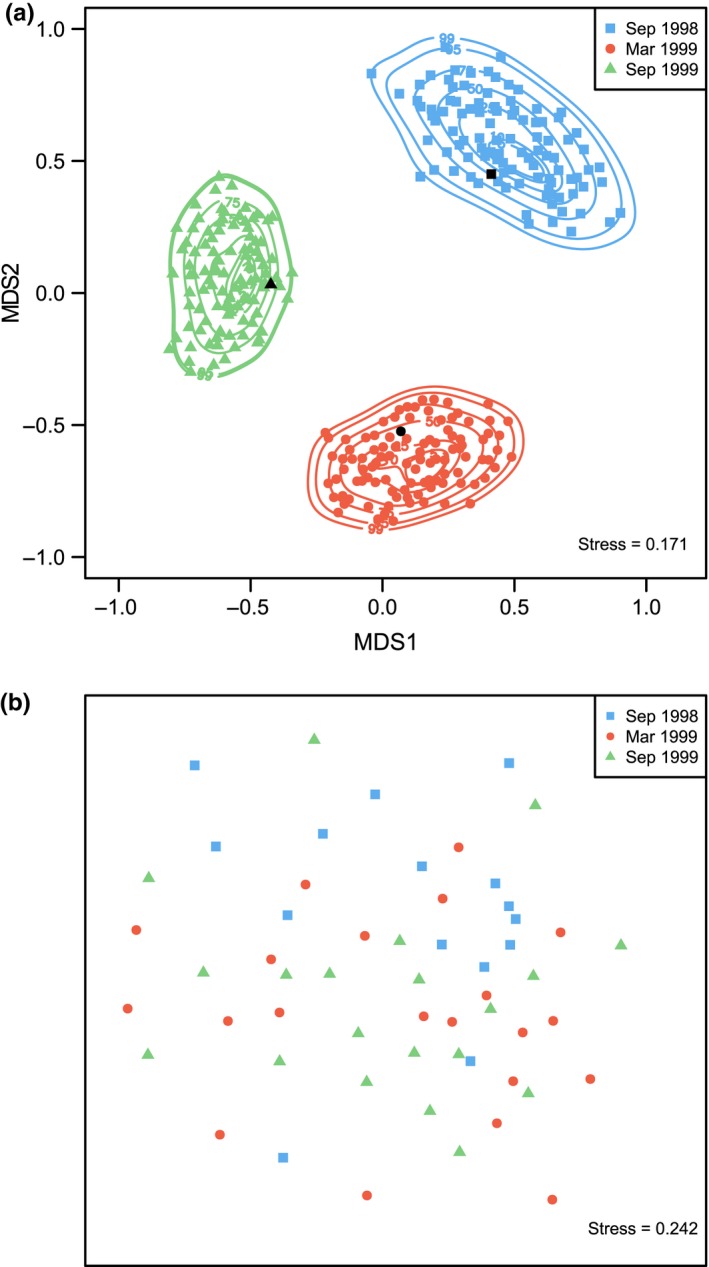
Ordinations based on Bray–Curtis dissimilarities of square‐root‐transformed abundances of fishes (47 species) from the Poor Knights Islands at three different times, obtained using: (a) metric multi‐dimensional scaling (mMDS) of distances among centroids for the original data (black symbols), also showing centroids of 100 datasets (colored symbols) generated under the full copula model (parameters estimated separately for each group, shown as three different colors) along with kernel density contours; and (b) non‐metric MDS plot of the original data, with replicate sites in each of three groups shown with three different colors

Using this approach for the Poor Knights’ dataset, the observed centroid for each group falls well within the distribution of centroids for that group generated under the copula‐based model (Figure 7a). The dispersion/shape of centroid distributions in the reduced‐space ordination also clearly varied among groups. This graphic is far more informative than the (typically drawn) non‐metric MDS plot of the original data (Figure 7b). The latter is dominated by large residual variation within each group and concomitant high stress, which masks group differences. Consider how, in the univariate analysis of data from ANOVA‐type study designs, one typically plots the means for each group, along with their associated standard errors (which sensibly assumes normality for the distribution of means under the central limit theorem), to visualize both the relative positions and the variation in the group means. Similarly, the ordination in Figure 7a depicts the centroid for each group (in the space of the chosen resemblance measure) along with a visualization of the multivariate variation in the positions of those centroids (shown as density contours) under the assumptions of the full copula model. By “full copula model,” we mean the set of estimated correlations among all pairs of species in the MVN copula space along with the full set of individual (in this case discrete) marginal distributions for each species and their associated estimated parameters.

**Figure 8:**
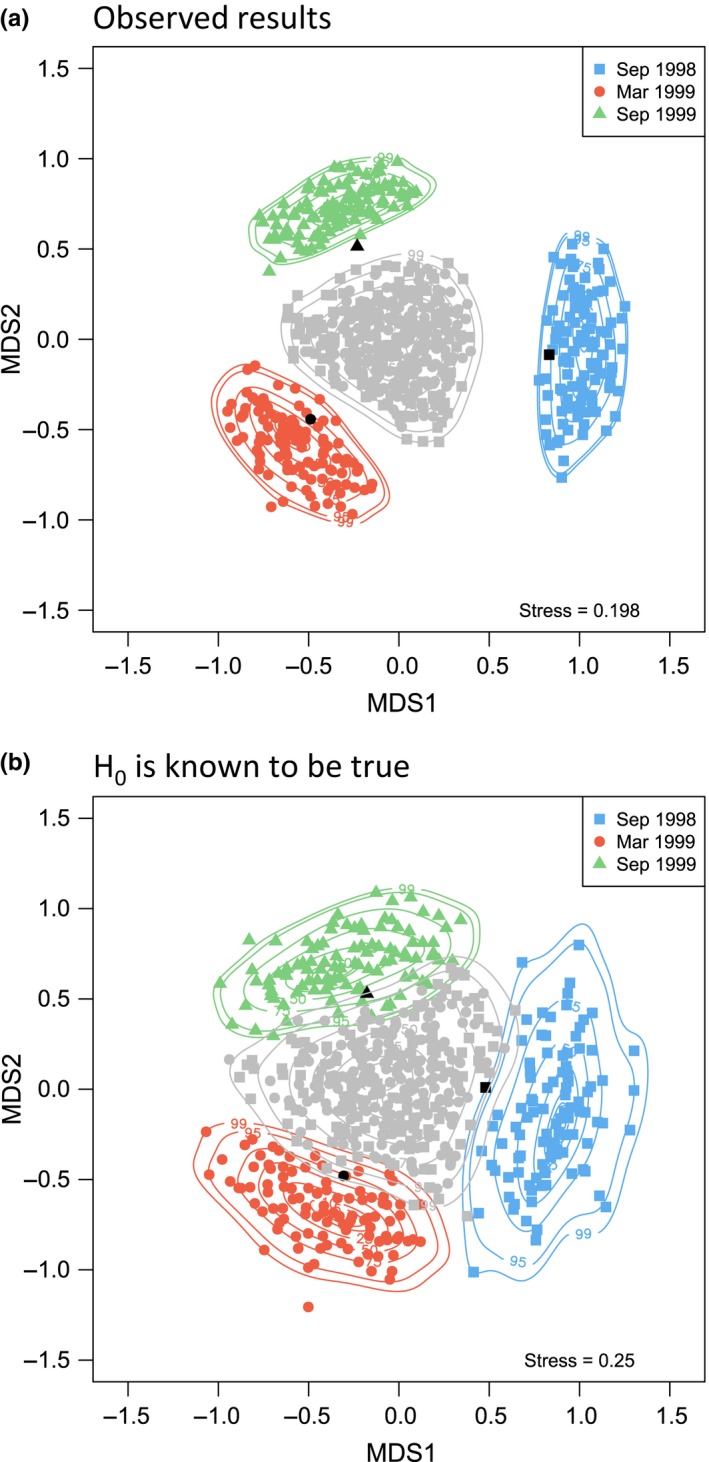
Ordinations based on Bray–Curtis dissimilarities of square‐root‐transformed abundances of fishes (47 species) from the Poor Knights Islands at three different times, obtained using: (a) metric multi‐dimensional scaling (mMDS) of distances among centroids for the original data (black symbols) along with centroids of 100 datasets generated under three separate copula models for each of the three groups (colored symbols, H_A_ is true) and centroids of 100 datasets obtained by random permutation of the sampling units among the three groups (gray symbols, H_0_ is true); (b) mMDS generated in the same manner as in (a), but where data consisted of “mock” observations where H_0_ was known to be true (see text for details)

It must be borne in mind, however, that Figure 7a was drawn under the assumption of a highly specific alternative hypothesis (H_A_). The three groups have been asserted to be different, and all of the model parameters (copula plus marginals) have been estimated separately for each group. It is therefore not surprising that these three regions do not overlap with one another in the ordination space. We might also choose to visualize distributions of centroids under a true null hypothesis (H_0_). For example, we can calculate centroids obtained under random permutation of the sampling units among the three groups. These permutation‐based centroids assert the null hypothesis that sampling units are fully exchangeable among the groups to be true. We can examine the distributions of centroids under H_0_ and also under H_A_ in a single ordination plot (Figure 8a). By including the originally observed centroids for each of the three groups here as well, we are able to gain an understanding of the position of our own data with respect to H_0_ and the specific H_A_ that arises from these copula models (Figure 8a).

One might well ask: what would such a plot look like if the null hypothesis were true? Specifically, suppose we do the following: (a) take the full set of *N* = 56 sampling units and estimate a single set of marginal and copula parameters from these data (acknowledging no a priori groups, so H_0_ is true); (b) generate three groups of “mock” data (with sample sizes of *n*_1_ = 15, *n*_2_ = 21, and *n*_3_ = 20) directly from that model, then treat this dataset as if it were our “observed” data, but here we know that H_0_ is actually true; (c) estimate marginal and copula parameters separately for each of these “groups” in our mock dataset; and then (d) simulate data and draw distributions of centroids under H_A_ and H_0_ in the same way as was done for Figure 8a.

For the mock dataset (Figure 8b), the distributions of centroids for the three groups generated under H_A_ (three different colors) do appear separate from one another. This is because they arose from three different sets of estimated parameters, even though the groups themselves, as we know, were, in this case, completely arbitrary. However, quite tellingly, the mock “observed” centroids (black symbols) also lie within the distribution of centroids under H_0_ (gray symbols). The overlapping of contours for centroid distributions drawn under H_A_ with those drawn under H_0_ also suggests a lack of real difference among the groups. Note too that there is quite high stress here (>0.24)—yet another signal that there is no distinctive group structure to display (Figure 8b). This can be contrasted with the clear group structure apparent in Figure 8a that was constructed based on the real data; we see no overlap in the centroid distributions under H_A_ with those under H_0_, and the positions of the genuine observed centroids clearly favor H_A_.

## 5 MODEL‐BASED INFERENCE AND POWER

### 5.1 Model‐based inference

**Figure 9:**
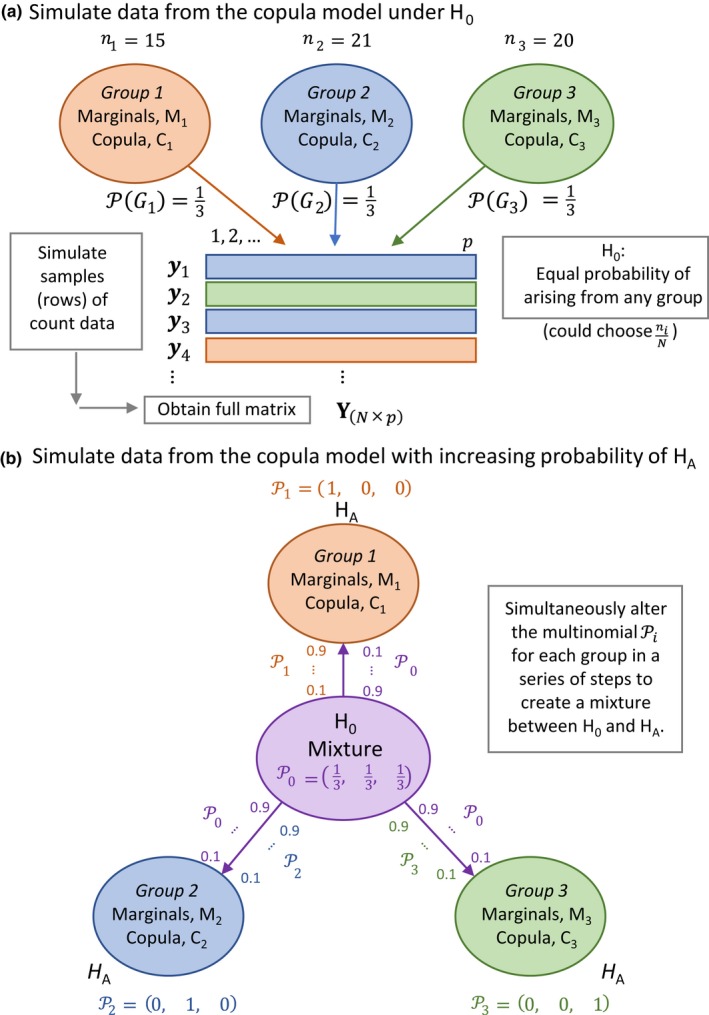
Schematic diagram showing methods of simulation using multinomial mixtures from copula models (with given parameters for the marginal, *M*, and copula, *C*, distributions) under (a) a null hypothesis of every sampling unit having an equal probability of arising from any of the groups; or (b) a specific alternative hypothesis for the case of three a priori groups of sampling units and where the alternative hypothesis asserts that all three groups are different from one another

We may generate data under a specified null hypothesis (H_0_) to achieve model‐based inference. One might consider a null hypothesis that asserts there are no groups and estimate a single set of parameters (marginals plus copula) for the full set of data. However, armed with a full copula model, having estimated separate parameters for each group, we may instead assume a simple null hypothesis that every sampling unit has an equal probability of arising from any group. Thus, we can generate **Y**_sim_ under H_0_ such that each vector ***y***_sim_ is drawn under a multinomial with probabilities of 1/*g* for each group (Figure 9a). For the Poor Knights’ data, these probabilities are $P_{i}=1/3$ for the *i* = 1, …, 3 groups. Another alternative would be to base null hypothesis probabilities on sample sizes: $P_{i}=n_{i}/N,$ appropriate, for example, if sample sizes directly reflected encounter rates of the existing groups in nature.

**Figure 10:**
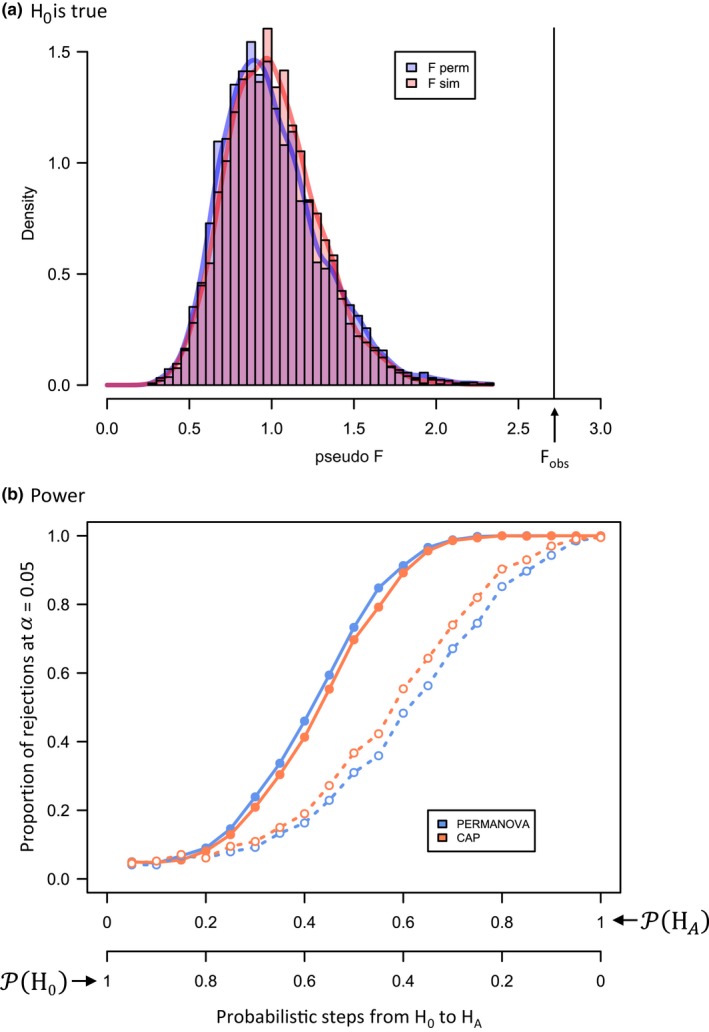
(a) Distribution of 5,000 values of the PERMANOVA pseudo‐*F* statistic to compare three times of sampling for the Poor Knights dataset based on Bray–Curtis resemblances calculated from square‐root‐transformed counts of *p* = 47 fish species obtained under permutation (F perm) or under the copula model (F sim); and (b) empirical power of PERMANOVA or CAP for 1,000 simulated datasets at each of 20 equal steps (as a multinomial mixture of probabilities between H_0_ and H_A_) for all 47 fish species (filled symbols) or for a subset of 16 fish species only that had estimated copula correlations of *ρ* ≥ 0.7 (open symbols). For each simulated dataset, *p*‐values were calculated using 999 permutations and seven principal coordinate axes were used for the CAP approach

With **Y**_sim_ generated in this way, one may calculate a test statistic, such as the PERMANOVA pseudo‐*F*, based on a chosen dissimilarity measure. Repeating this procedure many times generates a model‐based distribution of pseudo‐*F* under H_0_. A *p*‐value for model‐based inference is calculated directly as the proportion of pseudo‐*F* values under H_0_ that equal or exceed the observed value. In the present example, the observed value of pseudo‐*F* is 2.716 (vertical line in Figure 10a). A model‐based *p*‐value (from 4,999 random draws) is *p* = 0.0002. The assumptions here are that the specified copula and marginal distributions provide a realistic joint model for these data. The usual permutation‐based test of pseudo‐*F* is distribution‐free, so is preferable for robust inference (in this case, with 4,999 permutations, it yielded an identical *p*‐value to the model‐based *p*‐value); however, a close match between the model‐based distribution and the permutation distribution (e.g., Figure 10a) provides support for the validity of the model's assumptions.

### 5.2 Power analysis

Copula‐based models can be used to calculate power, thus to compare multivariate statistical tests under different scenarios. Power calculations require generation of data under a specified alternative hypothesis (H_A_). Sliding marginal parameter values (such as *μ*, *θ*, and/or *π*) between H_0_ and H_A_ separately for each species can produce unrealistic combinations of parameters. A smooth monotonic power curve is obtained, however, by modeling the continuum between H_0_ and H_A_ as a sliding scale of mixture probabilities.

Let P denote a *g* × *g* matrix of elements $P_{\mathrm{ij}}$, the probability of drawing a sample ***y***_sim_ for simulated group *i* (rows) from original group *j* (columns). For the Poor Knights’ dataset, we may consider matrices of probabilities under ${\text{H}}_{0}\;\left(P_{0}\right)$ and under ${\text{H}}_{A}\;\left(P_{A}\right)$ as:

$$P_{0}=\left[\begin{array}{ccc} 1/3 & 1/3 & 1/3\\ 1/3 & 1/3 & 1/3\\ 1/3 & 1/3 & 1/3 \end{array}\right]\;\mathrm{and}\;P_{A}=\left[\begin{array}{ccc} 1 & 0 & 0\\ 0 & 1 & 0\\ 0 & 0 & 1 \end{array}\right],$$

respectively (Figure 9b). Next, let *f_k_* be the fractional probabilistic distance from H_0_ to H_A_ in a chosen number of steps (*k* = 1, …, *n*_steps_), beginning with *f*_1_ =  0 when H_0_ is true and $f_{n_{steps}}=1$ when H_A_ is true (Figure 9b). Note that rejecting H_0_ is logically distinguishable from the assertion that H_A_ is true. Note also that a wide variety of H_A_ may be specified. At a given step *k*, the probabilities $P_{k}$ used to simulate data are as follows:

$$P_{k}=\left(1-f_{k}\right)P_{0}+f_{k}P_{\text{A}}$$

To provide an example, we generated power curves with *n*_steps_ = 20 and 1,000 simulated datasets per step for the Poor Knights’ dataset and compared the empirical power of PERMANOVA with canonical analysis of principal coordinates (CAP; Anderson & Robinson, 2003; Anderson & Willis, 2003). Tests were done using 999 permutations for each simulated dataset, and CAP analyses were done using seven principal coordinates (which maximized allocation success). When all variables were included, PERMANOVA was more powerful than CAP (Figure 10b); however, when only a subset of fish species having strong associations were included (namely, those having at least one association with an estimated *ρ* ≥ 0.70 in the copula model; there were 16 of these), then CAP was more powerful than PERMANOVA (Figure 10b). As an aside, we noted that generating power curves using a slightly different null hypothesis (i.e., where data under H_0_ were generated from a model having a single set of parameters estimated from the full set of *N* sampling units rather than being a multinomial mixture of equiprobable draws from three groups having three separate sets of parameters) made no substantive difference to any of the above results.

## 6 DISCUSSION

Copulas provide a rich and flexible approach for modeling associations among disparate types of variables. Recent advances in statistical methods to estimate parameters for copulas having discrete marginal distributions using MCEM (see Appendix 1 below) open new doors for modeling count data. By allowing marginal and copula parameters to vary over time, we uncovered a striking increase in the strengths of associations among fish species after the cessation of fishing at a no‐take marine reserve (Figure 5). Naturally, the ecological mechanisms responsible for generating these associations cannot be inferred from observational data alone, but would require additional investigations.

Generalized linear models, GLLVMs, and GJAMs all have tremendous potential for capably modeling count data, particularly if they are extended to allow for changes in over‐dispersion or zero‐inflation within a species, and changes in correlations among species in different habitats. They do, however, have a few natural limitations. GJAMs avoid using classical statistical distributions, but this comes at a cost—the utility of explicit count distributions for characterizing individual species as univariate variables is lost, and how to choose partition widths to accommodate different mean–variance relationships remains unclear. In GLLVMs, the relationship between each species and each latent variable is effectively linear on a log‐scale (when the default log link for count data is used), which may or may not be appropriate/desirable. Also, the latent variable mechanism for inducing correlations will affect estimation of individual species’ over‐dispersions (and vice versa), making these two conceptually distinct features difficult to disentangle.

Copula models can be readily extended to include other types of variables commonly encountered in ecology, such as biomass, percentage cover, ordinal data, or mixtures of these with counts. They share some of the desirable features of GLLVMs and GJAMs while presenting some distinct advantages. All three approaches can use an underlying MVN distribution to model associations, but copulas can also use other association models, accommodate a wider variety of parametric marginal distributions than GLMs, and do not entangle the association model with the marginal model. Although outside the scope of the present study, a natural next step would be to explore the predictive capabilities of GLLVMs, GJAMs and copula models across a broad range of ecological datasets.

An ecologically meaningful index of association between species (excluding joint absences) is well‐preserved by a Gaussian copula model. This has obvious immediate advantages, as methods to achieve parsimony in MVN models abound (Huang & Chen, 2015; Popovic et al., 2018). However, other types of copulas (Genest & Favre, 2007; Schölzel & Friederichs, 2008), including pair‐copula constructions (such as vine copulas, see Aas, Czado, Frigessi, & Bakken, 2009; Bedford & Cooke, 2001, 2002; Brechmann & Schepsmeier, 2013), and also non‐parametric methods (Iman & Conover, 1982), all deserve further exploration for their potential use in ecology.

The identification of appropriate marginal distributions for modeling abundances also deserves more study. For fish assemblages at the Poor Knights, changes in over‐dispersion and zero‐inflation through time were clearly evident (Supporting Information Table S2), highlighting the need for flexibility beyond typical exponential families used in GLMs. The relationship *σ* ^2^ = *αμ* ^β^ (“Taylor's power law”) is virtually ubiquitous for counts of any organism, with *α* and *β* being species‐specific (Kendal, 2004; Taylor, Woiwood, & Perry, 1978). Variance–mean relationships that follow a power law can be modeled using Tweedie distributions (Tweedie, 1984), a subset of exponential dispersion models (Jørgensen, 1997) or using contagious distributions (Douglas, 1980; Neyman, 1939) under a generalized Poisson model (Clarke, Chapman et al., 2006; Coly, Yao, Abrial, & Charras‐Carrido, 2016). Special cases of the generalized Poisson include the NB (Quenouille, 1949), Neyman Type A (Neyman, 1939), Pólya‐Aeppli (Kendall & Stuart, 1963), the discretized Poisson‐gamma (Foster & Bravington, 2013; Kendal, 2004), and the Poisson lognormal (Aitchison & Ho, 1989; Preston, 1948). Under‐dispersion, where *σ* ^2^ < *μ* (Rogers, 1974), which can occur for organisms exhibiting territorial behavior, or allelopathy (Rice, 1984), is also under‐studied. Copulas allow *any* marginal distributions to be used for individual species, including with zero‐inflation, facilitating broader and deeper investigations of this topic.

A fundamental question remains: What are the limits of our approach? For multivariate data, the available degrees of freedom (*df*), provided sampling units are independent, are likely to be bounded such that *N* ≤ *df *< (*N* × *p*), and to depend on the level of species’ inter‐associations. One alternative to our proposed preliminary screening for significant pair‐wise associations would be to identify subsets consisting of coherent groups of associated species (Somerfield & Clarke, 2013). These practical permutation‐based approaches may be complimented (or replaced) by direct regularization/shrinkage of the copula covariance matrix (Schäfer & Strimmer, 2005). One might also consider joint estimation of copula and marginal parameters. In any case, how to assess parsimony/model complexity in the full framework is an open question.

We have focussed on an ANOVA‐type study design. We chose to fit separate parameters (copula plus marginals) to data from each group. However, sampling units might, instead, occur along one or more measured environmental gradients. Continuous predictors can be included in marginal distributions for each variable (as in GLMs; Warton et al., 2015, Niku et al., 2017; Popovic et al., 2018). However, responses of species to gradients are generally unimodal and may be modeled this way (Jamil & ter Braak, 2013; Yee, 2004, 2015), either along each margin or potentially inside the Gaussian copula space. Associations would remain constant using such an approach; however, copula correlations themselves might also be modeled as a function of environmental variables (Nikoloulopoulos & Karlis, 2010)—an idea worth pursuing.

Our approach catered well to varying zero‐inflation, but did not optimize models of rare species. Rare taxa are difficult to model (Elith et al., 2006; Fithian, Elith, Hastie, & Keith, 2015) and may not occur randomly; some sites harbor greater coincidences of singletons (Ellingsen, Hewitt, & Thrush, 2007). SDMs can fail to capture the nature of inter‐specific associations reliably, particularly for organisms having low probabilities of occurrence (Zurell et al., 2018). Observational data are often too sparse to model rare taxa well individually, but richness (number of species per sampling unit) can be modeled as a Poisson (or Poisson‐binomial) random variable (Calabrese, Certain, Kraan, & Dormann, 2014; Gavish et al., 2017). Thus, future model developments could include richness as an additional response variable in a multivariate copula. Relationships between richness and abundances of prevalent species (or environmental variables) could be estimated, allowing potential clustering of rare taxa.

The proposed analysis pathway enables researchers to achieve a greater understanding of the roles and relationships among individual species, as well as providing a novel approach to ordination and power analysis for investigating community‐level hypotheses. A unique feature of this framework is that we do not consider model‐based methods (such as GLMs, GLLVMs, GJAMs, or copulas) as running counter to dissimilarity‐based methods (such as ANOSIM, MDS, PERMANOVA or CAP). Rather, they are complementary: It is not a case of “either, or,” but a case of “yes, and….” Probabilistic statistical models are essential for characterizing assemblages on a per‐species basis, including estimation of useful interpretable parameters (e.g., Supporting Information Table S2, Figure 5), and also for simulation and prediction. Added value clearly attends the casting of simulations from joint‐species models into dissimilarity spaces. Dissimilarity‐based methods integrate information across all species in a way that individual species‐based models do not. Fundamental ecological concepts such as proportions of species shared, turnover, beta diversity, variation in identities of species, or gestalt shifts in composition are all readily examined through the use of meaningful resemblance measures (Anderson et al., 2011; Anderson, Ellingsen, & McArdle, 2006; Clarke, Somerfield et al., 2006; Kraft et al., 2011; Legendre & De Cáceres, 2013). Ordinations that show not only relationships among centroids but also probabilistic variability in centroid positions (e.g., Figures 7a and 8a) are highly desirable. Moreover, the behavior of dissimilarity‐based tests, historically prized for their broad utility and lack of assumptions, can now be further explored under carefully formulated hypotheses articulated by formal joint statistical models (Figures 9b and 10b). By using the latest model‐based approaches in tandem with evolving community‐level approaches, as proposed here, we can draw the best from both worlds.

We consider that copula‐based joint models of species count data, particularly when combined with dissimilarity‐based tools, provide a rich new suite of flexible methods that will generate many new scientific insights in the analysis of ecological communities.

## AUTHOR CONTRIBUTIONS

MJA and PD conceived the ideas, PD contributed mathematical formulations for the MCEM and wrote Appendix 1, AP provided R code with contributions from PD and MJA, AEM proposed the idea of mixture models for power analyses, and MJA wrote the manuscript. All authors approved the final draft of the manuscript.

## Data Availability

Data consisting of counts of abundances of fishes from the Poor Knights Islands are provided as Supporting Information Table S1 and are also available from Dryad Digital Repository: https://doi.org/10.5061/dryad.3s6rm0f.

## APPENDIX 1

This appendix outlines the core idea of using a Monte Carlo Expectation Maximization (MCEM, Wei & Tanner, 1990) algorithm to estimate the covariance matrix for a Gaussian copula with discrete marginal distributions that is constrained to have unit variances along the diagonal (hence takes the form of a correlation matrix). By constraining the Gaussian copula covariance matrix in this way, our algorithm ensures that the variances of individual variables remain precisely as specified by their marginal probability mass functions. For other examples of Gaussian copulas for discrete data, see Dauwels, Yu, Xu, and Wang (2013) and, more recently, Popovic et al. (2018), who describe a general covariance modeling framework, including latent variable graphical models (Meng, Eriksson, & Hero, 2014) and sparse factor analysis (Carvalho et al., 2008).

### BASIC MCEM

The general setup for MCEM is as follows (see Dempster, Laird, & Rubin, 1977; Wei & Tanner, 1990). Let *y* be an observed data vector. Let *x* be latent states or “missing data.” Let *θ* be parameters. Define *f*(*y*|*x*; *θ*) and *f*(*x*; *θ*) to be the probability density (or mass) functions of *y* given *x* and *x*, respectively, as indicated by the arguments. The likelihood to maximize is as follows:

$$L\left(\theta \right)=\int f\left(y|x;\theta \right)f\left(x;\theta \right)d\theta$$

Define the maximum likelihood estimate we seek as

$$\hat{\theta }=\arg\max L\left(\theta \right).$$

The MCEM algorithm works as follows:

1. Start with some initial value *θ_k_*_ = 0_ of *θ*.
2. Draw a sample of many values *X* = {*x_i_*}, *i* = 1,…, *m*, from *f*(*x*|*y*; *θ_k_*).
3. Find *θ_k_*_ + 1_ as:

$$\theta_{k\;\text{+ 1}}=\arg\max\frac{1}{m}\sum_{i=1}^{m}\log\left(f\left(y|x_{i};\theta \right)f\left(x_{i};\theta \right)\right)$$

The Monte Carlo average is the approximation of:

$$\frac{1}{m}\sum_{i=1}^{m}\log\left(f\left(y|x_{i};\theta \right)f\left(x_{i};\theta \right)\right)\approx E_{x|y,\theta }\left[\log\left(f\left(y|x;\theta \right)f\left(x;\theta \right)\right)\right]$$

1. Repeat the previous two steps, which are known as the “*E*”xpectation step and the “*M*”aximization step.

Without the Monte Carlo approximation, it can be proven that, provided *θ_k_* is not a stationary point, iterations will always yield *L*(*θ_k_*_ + 1_) > *L*(*θ_k_*), so that *θ_k_* converges to $\hat{\theta }$, unless there are local maxima or other stationary points. With the Monte Carlo approximation, iterations will not converge to a single value, but will instead oscillate about $\hat{\theta }$, with a precision that depends on the number of Monte Carlo samples, *m*. Thus, the value of *m* should be determined by the required precision of the results. Operationally, the beauty of MCEM is that we maximize a simple average of log probabilities at each step. This is much easier to work with than the (log of the) expected value of the probabilities from the latent states.

### MCEM FOR DISCRETE GAUSSIAN COPULAS

In what follows, we shall think of each discrete observation as having an unobserved fractional component. Let *y_i_* = (*y_i_*_1_, …, *y_i_*_p_) be counts of *p* species from sampling unit (or “site”) *i*. Subscript *i* will be dropped in discussing the likelihood contribution for one site's data only. Let *f_j_*(*y_j_*) and *F_j_*(*y_j_*) be the marginal probability mass function and cumulative distribution function, respectively, for species *j*, assuming *y_j_* takes non‐negative integer values. Dependence on parameters, *θ*, is implicit. Let *f*(*y*) = ∏ *f_j_*(*y_j_*) and *F*(*y*) = [*F*_1_(*y*_1_), …, *F*_p_ (*y*_p_)]. Let *g*(*y*) be the joint density function defined using a copula. For a Gaussian copula, if the observations were continuous and *f_j_*'s were PDFs, this would be:

$$g\left(y\right)=\frac{\phi_{\Sigma }\left(z\right)}{\phi_{I}\left(z\right)}f\left(y\right)$$

where ϕ**_Σ_** is the multivariate normal density with zero means and unit variances and correlation matrix **Σ**, **I** is the identity matrix, and *z* = (*z*_1_, …, *z*_p_) is defined by $z_{j}=\Phi^{-1}\left(F_{j}\left(y_{j}\right)\right)$, *j* = 1,…, *p* where Φ is the standard normal CDF.

Let ${\tilde{f}}_{j}\left(y_{j}\right)$ be a probability density function defined by spreading the probability mass *f_j_*(*y_j_*) uniformly in the interval [*y_j_*, *y_j_* + 1); that means:

$$\tilde{f}\left(y\right)=f\left(\left\lfloor y\right\rfloor \right)$$

Now the discrete copula likelihood can be written as the following integral over a hypercube:

$$L\left(\Sigma |y\right)=\prod_{i}\int_{{\left[0,1\right]}^{p}}^{}\frac{\phi_{\Sigma }\left(\Phi^{-1}\left(\tilde{F}\left(y+u\right)\right)\right)}{\phi_{I}\left(\Phi^{-1}\left(\tilde{F}\left(y+u\right)\right)\right)}\tilde{f}\left(y+u\right)du$$

where *i* is for sites, and the integral is over *u* ∈ [0,1]*^p^*, where *p* is the number of dimensions.

To work in the space of the multivariate normal variables defined by *z*, we change variables to $z=\Phi^{-1}\left(\tilde{F}\left(y+u\right)\right)$, so $y+u={\tilde{F}}^{-1}\left(\Phi \left(z\right)\right)$ and

$$du=\frac{1}{\tilde{f}\left({\tilde{F}}^{-1}\left(\Phi \left(z\right)\right)\right)}\phi \left(z\right)dz.$$

This yields

$$L\left(\Sigma |y\right)=\prod_{i}\int \phi_{\Sigma }\left(z\right)dz$$

where the integral is over $z\in \Phi^{-1}\left(\tilde{F}\left(y+u\right)\right)$ for *u* ∈ [0,1]*^p^*. That is the region of *z* that corresponds to *y* + *u* falling in the interval [*y_j_*, *y_j_* + 1).

Another way to write this is by integrating over the entire range of *z* and using an indicator function to exclude *z* outside the range given above. This is written as

$$L\left(\Sigma |y\right)=\prod_{i}\int_{z}^{}\phi_{\Sigma }\left(z\right)I_{{\tilde{F}}^{-1}\left(\Phi \left(z\right)\right)\in \left[y\right.,\left.y+1\right)}dz$$

where *I*_condition_ is 1 if “condition” is true, and 0 otherwise. Now we are ready to view this in the MCEM framework:

1. The “latent variable” is *z*, whose probability density is *ϕ* **_Σ_** (*z*);
2. The parameters are the elements of **Σ**;
3. The “observed data” are the values of *z* falling in the valid range defined above. The probability of the observed data given *z* is $I_{{\tilde{F}}^{-1}\left(\Phi \left(z\right)\right)\in \left[y\right.,\;\left.y+1\right)}$;
4. We consider the marginal distribution parameters for *f* as given.

With these interpretations, *f*(*z*|*y*; **Σ**) (in the role of *f*(*x*|*y*;* θ*) in the section sub‐titled "Basic MCEM" above) can be sampled easily. For example, if we need to sample from z given *z* ∈ [*a*, *b*), we can make use of ϕ**_Σ_** in the relevant range. Then the MCEM algorithm would be:

1. Start with some initial value **Σ** *_k_*_ = 0_ of **Σ**.
2. Draw a sample of many values *Z *= {*z_i_*},* i = *1, …, *m*, from *f*(*z*|*y*; **Σ** *_k_*).
3. Find **Σ** *_k_*_ + 1_ as:

$$\Sigma_{k+1}=\text{arg}\;\text{max}\frac{1}{m}\sum_{i=1}^{m}\text{log}\left(\phi_{\Sigma }\left(z_{i}\right)I_{{\tilde{F}}^{-1}\left(\Phi \left(z\right)\right)\in \left[y\right.,\left.y+1\right)}\right)$$

Note that the Indicator function will always be 1 (because of how the *z_i_*'s were sampled). That leaves only the task of maximizing the log‐likelihood of a normal correlation matrix based on the “sample” of *z* values, given the previous value of the correlation matrix. For a general multivariate normal covariance matrix (having non‐unit variances along the diagonal and covariances in off‐diagonal elements), we can immediately write down the maximum likelihood estimator (MLE; Dwyer, 1967). However, to obtain the MLE for the correlation matrix, **Σ*_k_*_ + _**_1_, a closed‐form expression is not immediately available. We instead use a numerical optimization technique with a spherical parameterization of the correlation matrix (Pinheiro & Bates, 1996, see section 2.3 therein), which allows us to constrain variances to 1, correlations to the range (−1, 1), and ensures a positive‐definite result.

Repeat the previous two steps until a stopping criterion has been satisfied. A simple stopping criterion can be implemented by taking a set of the *n* most recent estimates of the parameters, **Σ** *_k_*_ −_ *_n_*_ + 1_, …, **Σ** *_k_*, calculating the log‐likelihood associated with each of those estimates, *ℓ*_1_, …, *ℓ_n_*, and fitting a simple linear model of *ℓ*_1_, …, *ℓ_n_* versus the integer values 1, …, *n* (corresponding to the *n* most recent iteration steps in the MC algorithm); the MCEM algorithm is terminated when there is no evidence against the null hypothesis that the slope parameter associated with this linear model is significantly different from zero (H_0_: *β* = 0). We consider a useful approach (avoiding type II error) will be to require *p* > 0.25 in order to assert that H_0_ is true. More efficient stopping criteria may be found by estimating the variance of parameters (Booth & Hobert, 1999) or their log‐likelihoods (Caffo, Jank, & Jones, 2005), using just the Monte Carlo samples from a single MCEM iteration.
